# Curvelet based automatic segmentation of supraspinatus tendon from ultrasound image: a focused assistive diagnostic method

**DOI:** 10.1186/1475-925X-13-157

**Published:** 2014-12-04

**Authors:** Rishu Gupta, Irraivan Elamvazuthi, Sarat Chandra Dass, Ibrahima Faye, Pandian Vasant, John George, Faizatul Izza

**Affiliations:** Department of Electrical and Electronic Engineering, Universiti Teknologi PETRONAS, Tronoh, Malaysia; Department of Fundamental and Applied Sciences, Universiti Teknologi PETRONAS, Tronoh, Malaysia; University of Malaya Research Imaging Centre, Kuala Lumpur, Malaysia

**Keywords:** Ultrasound image segmentation, Supraspinatus (SSP) tendon, Curvelet transform, Morphological operations

## Abstract

**Background:**

Disorders of rotator cuff tendons results in acute pain limiting the normal range of motion for shoulder. Of all the tendons in rotator cuff, supraspinatus (SSP) tendon is affected first of any pathological changes. Diagnosis of SSP tendon using ultrasound is considered to be operator dependent with its accuracy being related to operator’s level of experience.

**Methods:**

The automatic segmentation of SSP tendon ultrasound image was performed to provide focused and more accurate diagnosis. The image processing techniques were employed for automatic segmentation of SSP tendon. The image processing techniques combines curvelet transform and mathematical concepts of logical and morphological operators along with area filtering. The segmentation assessment was performed using true positives rate, false positives rate and also accuracy of segmentation. The specificity and sensitivity of the algorithm was tested for diagnosis of partial thickness tears (PTTs) and full thickness tears (FTTs). The ultrasound images of SSP tendon were taken from medical center with the help of experienced radiologists. The algorithm was tested on 116 images taken from 51 different patients.

**Results:**

The accuracy of segmentation of SSP tendon was calculated to be 95.61% in accordance with the segmentation performed by radiologists, with true positives rate of 91.37% and false positives rate of 8.62%. The specificity and sensitivity was found to be 93.6%, 94% and 95%, 95.6% for partial thickness tears and full thickness tears respectively. The proposed methodology was successfully tested over a database of more than 116 US images, for which radiologist assessment and validation was performed.

**Conclusions:**

The segmentation of SSP tendon from ultrasound images helps in focused, accurate and more reliable diagnosis which has been verified with the help of two experienced radiologists. The specificity and sensitivity for accurate detection of partial and full thickness tears has been considerably increased after segmentation when compared with existing literature.

## Introduction

Ultrasound is a medical imaging modality preferred by radiologist and physicians due to its capability to produce real time imaging data, reduced time in diagnosis, and high patient acceptability. Musculoskeletal (MSK) ultrasound is rapidly gaining importance for the assessment of joints and soft tissue disorders [1, 2]. Occurrence of MSK disorders are mostly found in sports, adulthood (>30 years of age) or in accidents due to sudden impact which leads to reduced functions of daily life.

**Figure 1:**
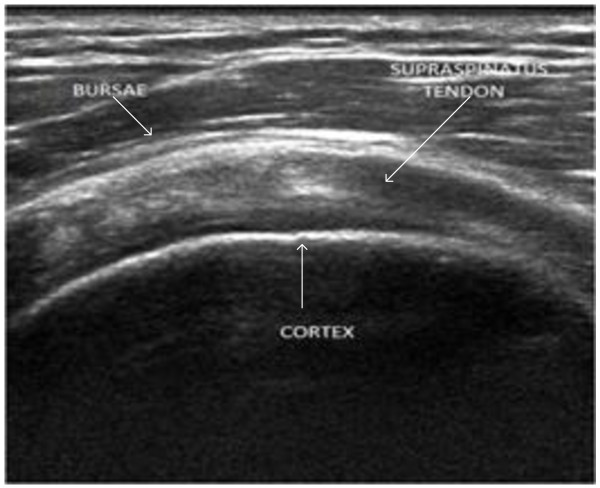
**SSP tendon ultrasound image.**

Supraspinatus (SSP) tendon is among the four muscles found in rotator cuff in shoulder; it runs from supraspinatus fossa superior of scapula to the greater tuberosity in humerus. Supraspinatus (SSP) tendon disorders are third most prevalent in MSK [3, 4]. The disorders in SSP tendon occur in the form of tear (loss of connective tissues binding collagen fibre), tendinosis (inflammation) and results in acute pain, insomnia and, reduced mobility of shoulder. SSP tendon as imaged using ultrasound equipment is shown in Figure 1. In ultrasound images, soft tissues such as muscle, fat, and other connective tissues reflect different echogenic pattern allowing the radiologist to differentiate between healthy and ailing tissue. Healthy muscle tissues have uniform and organized patterns which tend to absorb the ultrasound beam and appear hypoechoic compared to fat [5, 6]. However, tendons with pathological conditions are disorganized, diffused and have hypoechoic appearance compared to healthy tendons. Conventionally, health of tendons was visually examined based on image texture provided by ultrasound images which makes modality highly operator dependent. Recently, ultrasound applications are being trained to provide quantitative information about diagnostics of patient’s health and fitness [7–10]. To date, for supraspinatus tendon quantitative analysis is limited to cross sectional area and thickness calculation [11]. In one study [12], quantitative ultrasound techniques were employed wherein it computes structural measurements and mean echogenicity to discriminate between muscle pathologies and healthy conditions. In another recently published research, musculoskeletal ultrasound was used to quantify muscle kinematics during dynamic activities such as drop landing in healthy subjects [1]. Reliability study was conducted for the use of ultrasound as a quantitative information tool by a research group for inter-rater and intra-rater reliability, the study reveals that appropriately designed protocol will allow radiologist to identify structural changes within tendons [13, 14].

Despite growing interest and established techniques, few attempts are made to automate quantitative analysis of pathologies existing in tendon. In [15, 16], Horng *et al.* used feature extraction method based on gray level co-occurrence matrix (GLCM) and texture feature coding method (TFCM) to classify lesions in tendon. In [17], Nielsen *et al.* proposed method for tissue characterization using first order and higher order statistics for SSP tendon and thigh muscles. In [18], Michael *et al.* suggested a measurement method to estimate muscle dystrophy and sarcopenia via echogenicity measurement using grayscale analysis for musculoskeletal muscles. This work is focused towards automatic segmentation of SSP tendon from ultrasound image for focused analysis and serves as first step towards automated diagnosis of tendon.

Segmentation in ultrasound images can be difficult for numerous reasons such as contrast and resolution of image, speckle noise which is inherent property of ultrasound imaging modality, operator dependency of the modality. The paper addresses the issue of contrast enhancement, despeckling and issues occurring due to operator dependency for accurate segmentation of SSP tendon.

### Related work

Delineating boundaries and region of interest segmentation is a challenging task due to low contrast, resolution and inheritance of high intrinsic noise (speckle) in ultrasound images. Researchers [19–40] have suggested application specific segmentation technique for segmentation of ultrasound images. In [19], a brief review about ultrasound image segmentation techniques has been covered. The feature based segmentation techniques are often used in segmentation of ultrasound images. Image features such as gray level distribution [20], phase information [21], gradient information [22], shape of anatomical region [24] and temporal information have been exploited for accurate segmentation. In [24, 25], Rayleigh model has been extensively used in numerous occasions with anisotropic diffusion for removal of speckle noise and segmentation of ultrasound images. Rayleigh mixture model [26] was used for segmentation of skin lesions from ultrasound image. Rodtook [24] and Wang [27] have incorporated gradient information using active contour method and level set method for extraction of region of interest from ultrasound images. Intensity gradients are used along with anisotropic diffusion [28], morphological filtering [29], total variation filtering [30] and other filtering techniques [31, 32] for ultrasound image segmentation. Using shape priors is also very popular technique for segmentation of ultrasound images and has been used for segmentation of kidney [23], intravascular ultrasound (IVUS) [33], cardiac [34], and prostate ultrasound images [35]. Curvelet decomposition [36, 37] has recently been introduced in medical imaging for segmentation and enhancement of images. In [38], curvelet features are used for analysis of retinal images along with connected component analysis. Semi-supervised ultrasound image segmentation [39] technique is proposed using curvelet features and texture analysis. In [40], curvelet features are used in ultrasound images for segmentation of kidney images for better and enhanced diagnosis.

This is the pioneer work towards focused and automated segmentation of SSP tendon for accurate, focused and more reliable diagnosis. In this work, automatic segmentation of SSP tendon from ultrasound image is proposed. The method uses curvelet transform for feature extraction based on energy analysis of features followed by connected component analysis and morphological operations to accomplish the task.

### Material

The database of 116 images of SSP tendon ultrasound image with and without pathological condition was collected with the help of trained radiologist from Universiti Malaya Medical Centre. The data was collected over a period of three years with patients varying in age group from 19–54 years of age. The database was collected using Philips iU22 ultrasound and GE Logiqe BT2011 ultrasound machines. The database consists of images of patients with abnormalities such as single or multiples tears and tendinosis. 80 patients were observed to be suffering from only tendinosis, 100 patients were suffering from single or multiple tears in tendinosis or healthy tendon. The implementation of proposed methodology was done on MATLAB version 8.0.

### Proposed methodology

Ultrasound image of the SSP tendon when observed using database of collected images acquired by two different radiologists on two different machines with around 51 patients found to contain variations such as:

1. The width of the tendon,
2. Topography or location of tendon in images,
3. Intensity level of SSP tendon in image,
4. Radius of curvature of convex SSP tendon.

The observed differences are due to inter and intra-operator variability, acquisition system and patients anatomy. Therefore, for a method to be able to segment automatically the SSP tendon, it should be robust towards above variations. Despite above variations, invariants or unique features in ultrasound image of SSP tendon irrespective of machines, patients and radiologist are:

1. Convex nature of SSP tendon.
2. Compressed between bursae at the top and cortical bone at the bottom.
3. High intensity of bursae and cortical bone compared to nearby structures.

**Figure 2:**
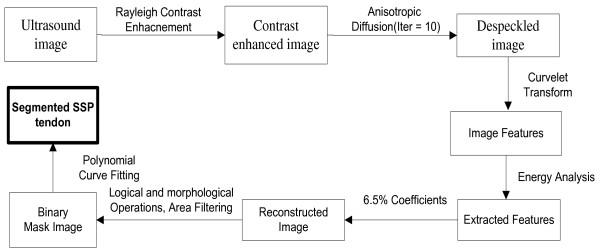
**Process flow for proposed model.**

Convex nature of SSP tendon which is evident from Figure 1, is found in all the images of the database. The location of SSP tendon runs from supraspinatus fossa superior of scapula to insert in greater tuberosity in humerus which makes it suppressed between bursae and cortex in all the images. Bursae and Cortex due to its high echogenic property exhibits hyperechoic structure compared to neighboring areas. These three unique features for SSP tendon were exploited to propose a robust method invariant to above variations for segmentation of SSP tendon. The process flow for proposed methodology for automatic segmentation of SSP tendon is shown in Figure 2.

Consequently, the proposed algorithm for automatic segmentation of SSP tendon follows the steps:

Step 1: Select ultrasound image of SSP tendon from database.

Step 2: Apply image enhancement using Rayleigh adaptive contrast enhancement followed by despeckling using anisotropic diffusion method.

Step 3: Decomposition of enhanced image by real valued curvelet coefficients using wrapping function.

Step 4: Morphological, Logical and Area filtering operation to remove the regions that do not belong to SSP tendon or are outliers.

Step 5: Polynomial curve fitting to smooth tendon and recover lost boundary points and construct the mask as per radiologist requirement.

The detailed methodology for proposed work is discussed in subsequent section.

#### Image enhancement and feature extraction

The ultrasound image suffers from poor resolution and contrast which causes traditional segmentation algorithm to poorly segment region of interest from the given image. In proposed method, ultrasound image obtained is first contrast enhanced. The contrast enhancement is performed using Rayleigh distribution following the concept that speckle in ultrasound image follows Rayleigh pattern [41]. The probability and cumulative density function of Rayleigh distribution is exploited to map the contrast enhanced pixel to image grid. The image pixels are mapped to the contrast enhanced image using the following equations.

Mapping for pixels in contrast enhanced image is performed using cumulative density function (CDF) of Rayleigh distribution which is described below
1

The probability density function (PDF) for Rayleigh distribution is given by,
2

The pixel wise mapping was done using,
3

where,
4

n_j_ gives the number of pixels in image with j_th_ gray level and *x* is the gray value. The value of σ for enhancement was empirically selected to be 0.4.

**Figure 3:**
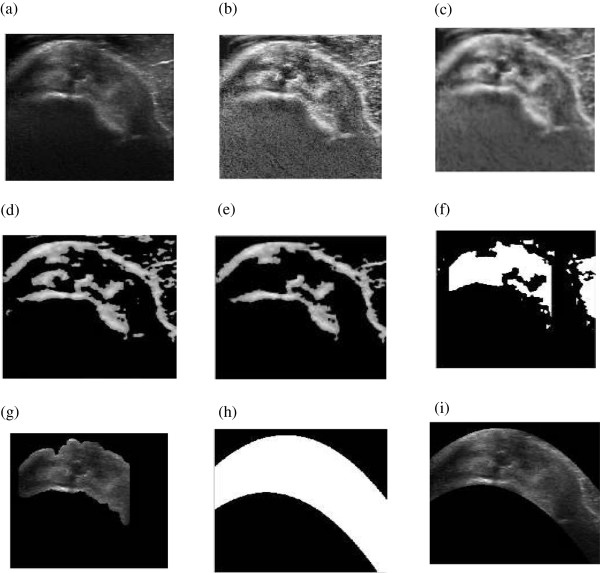
**Sequence of Steps for segmentation of SSP tendon (a) Original image (b) Contrast enhanced (c) Despeckled image (d) Curvelet transform (6.5% coefficient) (e) Area filtering (f) Morphological operations (g) Segmented tendon image (h) Polynomial interpolation (i) Final SSP tendon.**

Contrast enhancement makes the boundaries as well as speckle in ultrasound image more prominent. Therefore removal of speckle noise from the homogeneous region so as to keep the boundaries prominent is performed using anisotropic diffusion method [42]. Number of iterations of anisotropic diffusion performed for smoothing image is 10. The resultant image from contrast enhancement and despeckling is shown in Figure 3(b) and Figure 3(c) respectively. The preprocessing performed enhances the features needed for automatic extraction of tendon. The feature extraction from preprocessed image is performed using curvelet transform and is discussed in subsequent section.

#### Directional edge feature extraction

**Figure 4:**
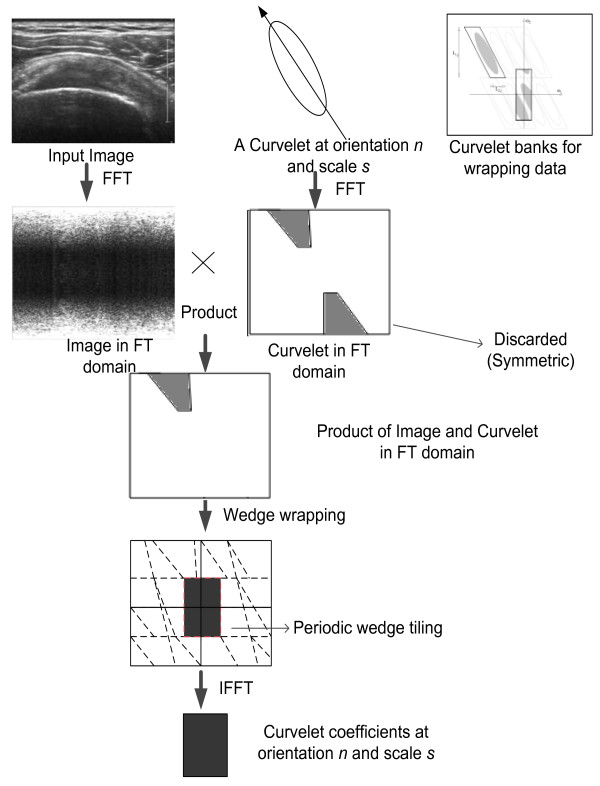
**FDCT via wrapping.**

The capability of curvelet transform to extract directional edge features at different orientations is exploited for extracting edge features from ultrasound image. Curvelet transform provides details such as spectral information successfully at different orientations with reduced complexity. Curvelet decomposition using wrapping function method and real coefficient is used because of reduced complexity and faster computation. The feature extraction from ultrasound image is performed using method illustrated in Figure 4. Detailed description about curvelet transform can be found in literature [37].

Curvelet transform decomposes the image in different direction and frequencies in the curvelet domain. Curvelet coefficients after decomposition are well adapted to contain edges from the image. SSP tendon ultrasound image consist of bright features, which comprise of Bursae and Cortex along with some outliers in the form of muscle fat mixture or abnormalities such as calcification. The brightest structures along with some outliers are likely to present in biggest curvelet coefficients after decomposition. To find best scale and percentage of coefficients, energy analysis is conducted at scales 2, 3, 4 and 5 with orientation 16 and 32. The percentage of curvelet coefficients were varied from 5 to 10. The method used for calculating the optimum number of features was based on the energy of the region delineated by radiologist. The energy of the region delineated by radiologist is correlated with the energy of the reconstructed image using curvelet coefficients at different scales and orientation.

**Figure 5:**
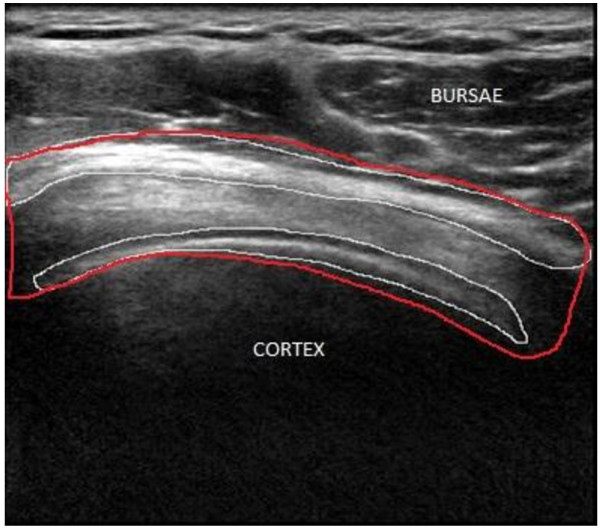
**Manual segmentation of Bursae and Cortex by radiologist.**

The manual delineation of SSP tendon was performed by radiologist and is shown in color outline in Figure 5. The segmentation region includes bursae and cortex. The biggest curvelet coefficients contains region with highest intensity values which in this case happens to be: bursae, cortex and outliers. The idea is to filter out coefficients of high intensity values and remove outliers generating a mask to segment SSP tendon. The energy of manually segmented bursae and cortex by radiologist was calculated using the formula

5

where, p(i, j) is the pixel intensity at (i, j) and m* n are the total number of pixels in an image. The fraction of the energy contained in the area segmented by radiologist is computed. The ratio is calculated for the amount of energy contained in manual segmented region to that of total energy of the image.
6

The amount of energy contained in region extracted by radiologist was found to be consistent at 40 ± 5%, with almost all the images of the database. The best scale and orientation from curvelet decomposition was selected based on the energy analysis performed over scales: 2, 3, 4 and 5 with different orientation. The results of the analysis are shown in Table 1.

**Table 1:** **Results with varying percentage of coefficients at different scales and orientation**

	Percentage of curvelet coefficients (%)										
Scales/orientation	5	5.5	6	6.5	7	7.5	8	8.5	9	9.5	10
2/16	54.7	57.6	61.4	64.06	69.0	70.6	73.1	74.68	82.2	80.9	82.8
2/32	54.7	57.6	61.4	64.06	69.0	70.6	73.1	74.68	82.2	80.9	82.8
3/16	95.83	96.64	98.8	99.10	99.1	99.11	99.0	99.2	99.5	99.7	99.8
3/32	97.4	97.4	98.6	99.4	99.8	99.4	99.3	99.3	99.6	99.8	99.7
4/16	96.8	96.43	97.0	97.78	98.5	98.8	98.9	98.91	99.4	99.47	99.6
4/32	97.7	97.3	98.6	98.1	97.9	98.2	98.0	98.8	99.2	99.4	99.3
5/16	95.18	95.7	95.7	96.3	96.5	97.4	97.5	98.4	98.8	98.9	99.1

Table 1, shows the ratio of energy when 5%-10% of the curvelet coefficients are used to reconstruct ultrasound image. The ratio is calculated between the image reconstructed using varying percentages from 5%-10% to that of total energy of the image. It suggests that scales 3, 4, 5 at different orientation provides energy ranging from 95%-99.9% with curvelet coefficients percentage from 5%-10% respectively.

From the analysis performed above, on segmented image by radiologist, it has been found that bursae and cortex together contains 40 ± 5% of energy. Therefore, the decomposition of curvelet at higher scales was avoided. The decomposition at scale 2 with orientation 16 and 32 was performed, as evident from Table 1, and it is found that the amount of energy contained, when two different orientations are chosen is almost same. Therefore, in order to save computational time scale 2 with 16 orientations were chosen. The energy level increases with increasing percentage of coefficient from 5-10%. The image reconstructed with percentage coefficient 6.5%-7% was found to contain the region manually segmented by radiologist along with outliers in the form of muscle fat-mixture.

**Figure 6:**
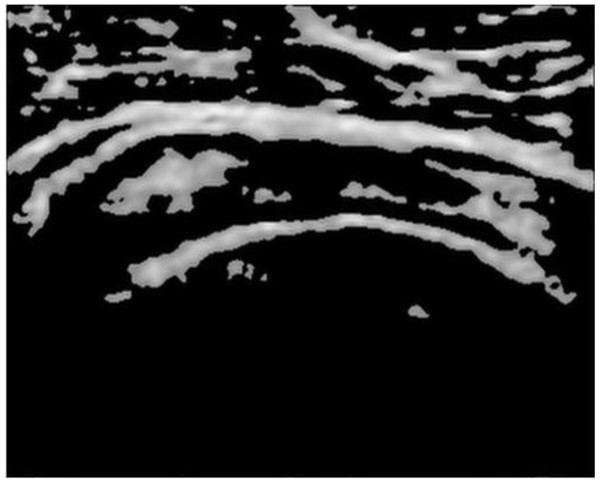
**Image reconstructed with 6.5% of curvelet coefficients at scale 2 and 16 orientations.**

The reconstructed images with 6.5% curvelet coefficient are shown in Figure 6 and Figure 3(d). The image contains several outliers that are unavoidable along with bursae and cortex. If lower percentage of coefficients is chosen, then, the structure for bursae and cortex starts to deteriorate. The reconstructed image with 6.5% curvelet coefficients contains 64.06% of the energy, wherein the bursae and cortex along with approximately 15%-20% of outliers is found. The energy test was conducted on several images from dataset and the results were found to be consistent. The second level with 16 orientations was found to be giving the best approximation for energy and was closest to radiologist’s segmentation. The increase in the energy of the reconstructed image is consistent with the outliers that are generated in the process to automate the segmentation. The next step of the algorithm focuses on removing the outliers. In the subsequent steps, the removal of outliers is performed using mathematical concepts of morphological operations and area filtering.

#### Area filtering and connected component analysis

The outliers in the reconstructed image remain in the form of bright structures which could be either muscle fat mixture or some form of an abnormality, i.e. calcification. The reconstructed image is rescaled to gray levels (0 ~ 255) to perform connected component analysis and thresholding. The removal of intensity components with lower intensity levels is performed by applying threshold based on formula introduced in [43] that is,
7

where, μ is the mean and *σ* is the standard deviation respectively. The value of α (*α* < 1) is chosen small enough. The thresholding is performed based on
8

where, *p*(*i*, *j*) is the value of pixel intensity at location (*i*, *j*) in an image. To remove undesired pixels or blobs from image area filtering is applied. To perform this, connected component analysis (CCA) with eight neighborhood is performed where the connected pixel component that are identified below certain threshold are removed. The threshold value is specified empirically, which involves analyzing the number of pixels in bursae or tendon for reconstructed image. From the analysis of more than 100 images with the size (421x580), it has been found that blobs with total number of pixels values less than (~ < 5000) tend to be the outlier. The thresholding value is relative to the size of the image. The image formed after the thresholding is shown in Figure 3(e).

Although most of the undesired objects are removed in this step, but some of the components connected with bursae and tendon are still inevitable. Therefore, desired mask for segmentation of SSP tendon is obtained by removal of these remaining outliers.

The dilation, erosion, opening, closing are standard mathematical morphological operations that helps in the removal of distorted texture and noise from image using structure element. For this application, disk was used as structure element. Morphological operations leave the structures bigger than SE unchanged. The drawback of conventional dilation and erosion is that they do not preserve edge information perfectly. The new operator proposed by Bangham *et al.*[44], takes care for above drawback and emphasizes on size of structure but forgets the shape completely. Morphological operators that take care of above problem and consider both shape and size were proposed [45]. If the image is I(x, y) and g is the mask image, then general equation for geodesic dilation is defined by equation
9

And, the equation for geodesic erosion is written as
10

Step by step execution and pointwise application of maximum (minimum) operator after each iteration of elementary geodesic erosion (dilation) is necessary to control the marker image. In general, after execution of some steps both the above equations becomes stable and no further changes occur that means
11

based on above definitions, opening and closing operation by reconstruction is performed as below:
1213

**Figure 7:**
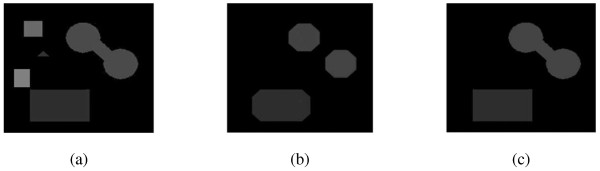
**(a) Original image (b) Result of conventional opening (c) Result of opening by reconstruction.**

Application of conventional morphological operations sometimes introduces new edges and contours which are not needed. Reconstruction by geodesic morphological operations yields results wherein these drawbacks are removed. Sample test of geodesic morphological operations is shown in Figure 7. The morphological operation shown uses disk as the structure element.

The morphological operation is performed to remove the remaining objects. The method involves two steps: one is morphological dilation followed by erosion operation. The structure element used for morphological operations is a disc with the size 17x6. The result obtained from morphological operations is shown in Figure 3(f). In the final step, the area filtering is applied wherein the high threshold value is chosen so as to obtain the mask for SSP tendon. The sequence of steps for image segmentation is shown in Figure 3.

Therefore, the algorithm is scripted as follows:

1. A thresholding operation is performed to remove high intensity values. The threshold value is chosen based on Eq. (27).
2. Connected component analysis (CCA) with 8-neighborhood is performed for removal of blobs not belonging to SSP tendon which are considered as outliers.
3. Area filtering is performed to remove the blobs with small number of pixels which do not belong to tendon.
4. Morphological operation of opening is performed using structure element of 17×6.
5. In last step, again area fileting is used to obtain the clear structure of tendon.

The results at this stage, shown in Figure 3(g), were presented to radiologist for assessment. After discussion, recommendation was put forward to extract smooth boundary for SSP tendon which enclose bursae and cortex.

#### Polynomial curve fitting and morphology

The boundary for extracted mask was found and polynomial curve fitting is applied to make the boundary of the mask smooth for better visualization of the tendon. Two different quadratic functions were estimated based on the boundary point of the extracted region. The curve fitting was performed by using quadratic polynomial function. The result for this step is shown in Figure 3(h).

The curve fitting also recovered some of the missing boundary points from images thereby increasing the accuracy of the results. The experimental results of proposed algorithm and radiologist assessment of segmentation is discussed in next section.

### Performance evaluation

**Figure 8:**
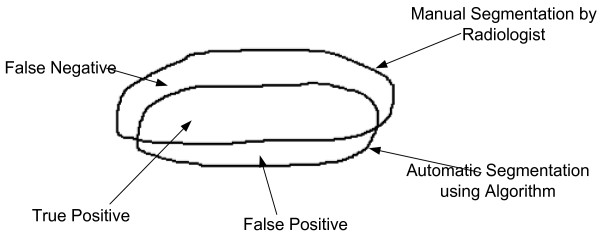
**Corresponding areas for false positive, false negative and true positives.**

The proposed algorithm was assessed based on the quantitative and qualitative analysis. The quantitative analysis for the assessment of proposed methodology for segmentation was assessed using three metrics: 1) false positive rate (FPR); 2) true positive rate (TPR) [46]; 3) Accuracy (ACC). The radiologist were requested to delineate SSP tendon manually ultrasound image. The comparison of the area delineated by radiologist and automatic segmentation performed by proposed algorithm was done based on the area of true positive, false positive, true negative, false negative. The Pictorial representation for corresponding area is shown in Figure 8.

The formula used to calculate FPR, TPR and Accuracy is,
141516

where , A_m_ is the manual segmentation for area of SSP tendon performed by an experienced radiologist. A_a_ is the automatic segmentation of SSP tendon performed by proposed methodology.

In the second round of assessment, the segmented images were provided to radiologist and blind evaluation was performed for partial thickness tear (PTTs) and full thickness tear (FTTs). And the results obtained by radiologist assessment were modelled so as to give sensitivity and specificity value for the method. The true positives, false positives, false negatives and true negatives in this were defined as

True Positives- pathology existent and diagnosed correctly

False positives- pathology not present and diagnosed

False negatives- pathology present but not diagnosed

True Negatives- pathology not present and not diagnosed

The sensitivity and specificity were calculated using the formula below
1718

The results for assessment of pathological condition were computed and compared with the diagnosis of PTTs and FTTs using ultrasound images.

### Experimental results

**Figure 9:**
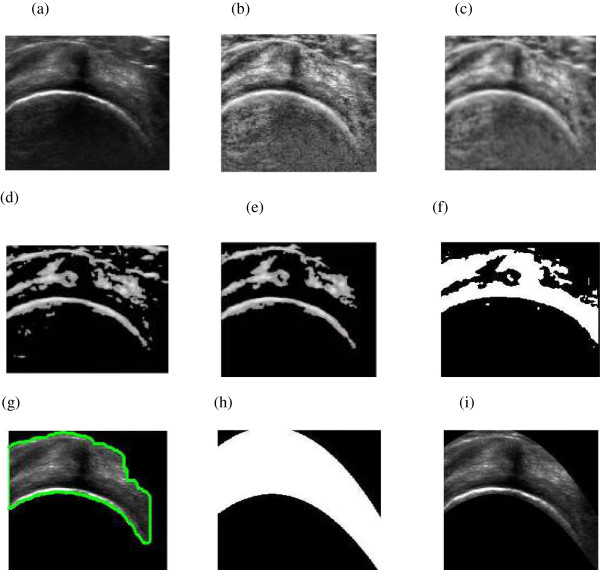
**Stepwise results of proposed method (a) Original image (b) Contrast enhanced (c) Despeckled image (d) Curvelet transform (6.5% coefficient) (e) Area filtering (f) Morphological operations (g) Segmented tendon image (h) Polynomial interpolation (i) Final SSP tendon.**

The result of tendon segmentation as per radiologist assessment was performed with the set operations described in proposed methodology section. The results of each step are shown in Figure 9.

**Figure 10:**
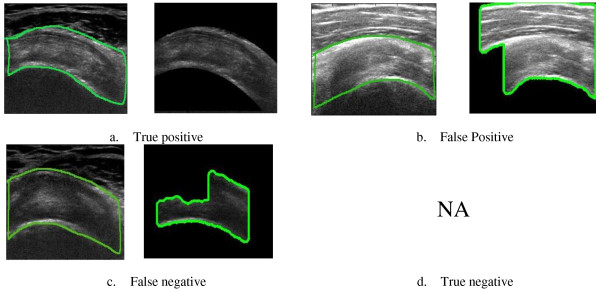
**Results from manual segmentation of radiologist and automatic segmentation using proposed algorithm (a) True positive (b) False positive (c) False negative (d) True negative.**

Figure 10, shows qualitative comparison of the results using manual segmentation by radiologist and result obtained using proposed algorithm. Figure 10(a) shows the true positive results wherein accurate segmentation as per radiologist requirement was attained. Figure 10(b) it can be seen that the region segmented by radiologist is present in the result with an outlier region shown. The false positives arise because of the inaccurate detection of muscle fat outlier which plays important role in segmentation. In Figure 10(c), result shows inaccuracy of the algorithm in the form of detection of false negatives, wherein the reason for inaccuracy is poor visibility of bursae. No cases of true negatives were found (true negatives are cases when the segmentation area completely lies outside the region manually segmented by radiologists). The quantitative assessment of results obtained using proposed algorithm was performed using the above mentioned parameters.

Table 2 shows the accuracy of the results in the form of true positive rate, false positive rate and accuracy of the result.

**Table 2:** **Performance result of the algorithm**

Performance metric	Evaluation result
TPR	0.9137
FPR	0.0862
ACC	0.9561

The performance of proposed method was also tested for focused assessment with the help of two radiologist and result were compared with the existing literature [47–49]. The results were compared for the diagnosis of partial thickness and full thickness tear using sensitivity and specificity values.

The results of comparison are shown in Table 3. In [47], authors used 40 images to compute the sensitivity and specificity of the diagnosis of PTTs and FTTs in SSP tendon, whereas Singh *et al.* in [48] uses 36 images to quantify the diagnosis of PTTs and FTTs in SSP tendon and Rutten *et al.* in [49] uses a database of 68 images to quantify the diagnosis of PTTs and FTTs in SSP tendon using ultrasound images. In this work, authors have used a set of 116 ultrasound images to qualitatively evaluate the performance of proposed method for assessment of PTTs and FTTs in ultrasound images. The sensitivity and specificity values for PTTS computed using [47, 48], and [49] are 92.3% and 92.6%, 66.7% and 93.5%, 92% and 33.5% whereas using the proposed method the values are 94% and 93.6%. Similarly, for FTTs sensitivity and specificity for [47, 48], and [49] are 92.6% and 94%, 92.3% and 94.4%, 94% and 94% resp. whereas sensitivity and specificity for diagnosis of FTTs using segmented SSP tendon are 95.6% and 95% respectively.

**Table 3:** **Comparison of results for focused pathology (PTT- Partial thickness tear; FTT- Full thickness tear)**

Author	Patient	Partial thickness tear		Full thickness tear	
		Sens.	Spec.	Sens.	Spec.
E. ElGawad *et al.*[47]	40	92.3	92.6	92.6	94
Singh *et al.*[48]	36	66.7	93.5	92.3	94.4
Rutten *et al.*[49]	68	92	33.5	94	94
**Prop. Method**	**116**	**94**	**93.6**	**95.6**	**95**

Algorithm run-time for automatic segmentation of SSP tendon is 1.4 s. The algorithm was implemented using i7, 3.4 GHz processor and 8GB RAM. The enhancement of image for segmentation takes 0.55-0.65 s and segmentation part takes about 0.75-0.85 s. Therefore, the proposed approach is fast, reliable with scope of possible future application in real time diagnosis. The assessment parameter and computation time for the algorithm suggests high performance for proposed methodology.

### Transportability of method

Ultrasound is a deterministic imaging modality which means images acquired under identical circumstances will yield similar result. Despite being deterministic in nature ultrasound image is highly operator dependent. In the proposed method, the attempt has been made to decrease the operator dependency of ultrasound machine for diagnosis of pathological conditions in SSP tendon. The image features are studied from images taken from different operator and machines and analyzed for unique features. The method uses image processing methods to extract features from the tendon and reconstruct SSP tendon. In all 116 images taken from different source and two different radiologists it was found that SSP tendon is convex in nature and is always compressed between bursae at the top and humeral cortex at the bottom. Both bursae and cortex have hyperechoic texture as compared with tendon. Since proposed method is based on the redundant nature of occurrence of SSP tendon in ultrasound image and the algorithm is tested for variability in equipment and operators visualization of SSP tendon. Therefore, proposed method can be used in clinical settings for post processing of SSP tendon ultrasound image for pathologies in SSP tendon with reduced inter and intra observer variability. The method can also be effectively used for training of medical officers for focused and effective diagnosis of SSP tendon pathology. The future work is intended to reduce the complexity of algorithm so that it can be effectively used as real time imaging tool embedded in ultrasound equipment for clinical analysis of SSP tendon.

### Discussion and future work

Before starting the research, literature survey on the existing methods for diagnosis of SSP tendon was studied. Also, several rounds of discussions with radiologist were performed regarding the state of art methods for diagnosis of SSP tendon. The problems faced by patients and radiologist during examination were consulted. It was found that MSK ultrasound techniques provide subjective evaluation regarding existing pathologies in tendon and are also painful because of duration of examination. The initiative was taken to automate the pathologies in SSP tendon. To further the research and automate pathologies first challenge was to locate region of interest i.e. SSP tendon automatically so that focused and accurate diagnosis for the ailment can be performed.

In this paper, a novel method for automatic segmentation of SSP tendon from ultrasound image is proposed. The method involves image enhancement and feature extraction from ultrasound image. The image was contrast enhanced using statistically adaptive method followed by speckle removal using anisotropic diffusion method. The image was then decomposed using curvelet transform. The energy analysis of decomposition was performed to select the amount of curvelet features needed for mask generation. It was found, that 6.5% of curvelet features, at scale 2 and 16 orientations, provides best mask for segmentation. Images were reconstructed using extracted curvelet features and geodesic morphological operations were used to extract edges and remove outliers. Connected component analysis and area filtering were applied to remove the remaining false areas and perform accurate detection. There is a trade-off between selecting curvelet features and removal of false areas. High percentage of curvelet features results in increase of false positives. The polynomial curve fitting is used to smooth the area of SSP tendon as per radiologist’s recommendations. The segmented SSP tendon will assist the radiologist for focused and accurate diagnosis of abnormalities in the tendon. The quantitative assessment performed for segmentation and results of diagnosis for pathological conditions suggests the effectiveness of proposed algorithm. Also the computation time for algorithm shows the capability of the algorithm to be made available for real time diagnosis of pathologies in SSP tendon.

In future, the work will be focused to provide an automated system for pathology in SSP tendon. The computation time of the algorithm will be reduced by refining extracted coefficients so that possible implementation in real time diagnosis is possible.

## Conclusion

The automatic segmentation of SSP tendon was successfully achieved and radiologist assessment for segmentation was performed. As per radiologist comments, the results help in enhancing the accuracy of diagnosed pathology because of focused assessment of tendon. The accuracy for the assessment of segmentation of SSP tendon is 95.61%. When diagnosing tendinosis, tear or calcification in SSP tendon segmented tendon provide focused and more reliable result with increased sensitivity and specificity. The proposed algorithm well suited for real time applications for musculoskeletal ultrasound in SSP tendon.
